# Update on Ventricular Tachyarrhythmias and Related Sudden Cardiac Death

**DOI:** 10.1016/s0972-6292(16)30825-7

**Published:** 2014-12-15

**Authors:** Johnson Francis, Narayanan Namboodiri

**Affiliations:** 1 Baby Memorial Hospital Ltd., Kozhikode, Kerala, India; 2 Sree Chitra Institute for Medical Sciences and Technology, Trivandrum

**Keywords:** Ventricular Tachyarrhythmia, Sudden Cardiac Death

This section on 'EP Update - Clinical' summarizes the recent literature on ventricular tachyarrhythmia and related sudden cardiac death from leading journals. The articles have been hand picked and reviewed by the editors of IPEJ for the benefit of readers.

## Risk of sudden cardiac death in nonischemic dilated cardiomyopathy

Goldberger JJ and colleagues have provided a meta analysis on risk stratification for arrhythmic events in nonischemic dilated cardiomyopathy [1]. They identified 45 studies with over six thousand participants which looked at arrhythmic events and predictive tests [Table 1]. They found that the tests of autonomic variability like heart rate variability, heart rate turbulence and baroreflex sensitivity were unable to predict arrhythmic outcomes to a significant extent. Other tests which include functional parameters, arrhythmic markers and depolarization / repolarization abnormalities have only a modest value in prediction of risk of sudden cardiac death in nonischemic dilated cardiomyopathy. Authors suggested that a combination of tests will be needed for optimal risk prediction in nonischemic dilated cardiomyopathy. This may be because multiple factors are involved in the pathogenesis of arrhythmia in nonischemic dilated cardiomyopathy, unlike in ischemic cardiomyopathy. Risk stratification is of paramount importance on deciding who should receive the limited and costly resource of an implantable cardioverter defibrillator. Ideally a good risk stratification method should give an odds ratio of more than 15, while most of the present risk stratification methods give an odds ration in the range of 2. It is also worth noting that most sudden cardiac deaths occur in those who cannot be identified by current risk stratification methods.

## Evaluation of pediatric relatives of sudden arrhythmic death victims

Sudden arrhythmic deaths occur in the range of about one in a hundred thousand population. Identifying inherited cardiac conditions in this group will be of benefit to the family members in prevention of future catastrophes. Wong CH et al [2] looked at the data from two centers on pediatric relatives with family history of sudden arrhythmic deaths. They identified 112 pediatric relatives from 61 families. Of the evaluated pediatric relatives 6 had inherited disorders and all of them were first degree relatives. Only 12 lead ECG, exercise testing and ajmaline provocation tests were found to be useful in the diagnosis of these inherited cardiac conditions. This was among an array of investigations including transthoracic echo, signal-averaged ECG, Holter monitoring, exercise testing, cardiac magnetic resonance imaging and genetic testing when indicated as per clinical guidelines. In this study samples were not available for molecular autopsy. Authors have also proposed a management pathway for these children. It may be noted that children without evidence of inherited cardiac disorders at initial evaluation also need follow up as all disorders may not manifest at young age.

## Higher risk of ventricular tachyarrhythmias and poor reverse remodeling in CRT patients with atrial ventricular ectopy

It is well known that highest percentage of biventricular pacing is optimal for patients on cardiac resynchronization therapy (CRT) for heart failure. Ruwal MH and associates [3] have studied the influence of atrial and ventricular ectopic beats in pre-implantation Holter monitoring on the outcome of CRT as well as the potential for ventricular tachyarrhythmias. They noted that ectopic beats increase the chance of lower biventricular pacing, less reverse remodeling, higher risk of heart failure, death and ventricular tachyarrhythmias. We are aware that premature ventricular complexes (VPC) can cause or worsen heart failure. VPCs can also lower the probability of super response to CRT. Due to the abnormal sequence of activation, VPC causes dyssynchrony by itself. PVC by inhibiting a paced beat can reset the post ventricular atrial refractory period so that the next sinus beat may not be sensed and not trigger biventricular pacing. Atrial premature beats can be associated with unrecognized atrial fibrillation which can impair CRT function due to the irregular RR interval. Irregular RR intervals can also increase sympathetic activity and enhance the chance of ventricular tachyarrhythmias and cardiac arrest.

## Early recurrence of ventricular tachycardia after catheter ablation in those with structural heart disease

Ventricular tachycardia (VT) associated with structural heart disease is often difficult to treat by catheter ablation. Recurrence of arrhythmia after ablation is associated with increased mortality. Nagashima K et al [4] found that one fifth of patients undergoing catheter ablation of VT in association with structural heart disease have an early recurrence within a week, most of it being in the first two days. Early recurrence of VT increased the long term mortality risk more than two and a half times. Programmed stimulation after ablation had limited role in prediction of recurrence as more than two third of those with inducible VT at the end of the procedure had no recurrence. But more than half of those who had a recurrence had inducible VT at the end of the procedure. On the other hand 17% of the 211 patients who had no inducible VT at the conclusion of the ablation procedure had early recurrence of VT. This is attributed to healing of the ablation lesions. VT morphology was similar to that of the initial clinical VT in half of the recurrences. Reduction of antiarrhythmic drug dose might have contributed to recurrence in 40% of the early recurrences. Early recurrence of VT was in independent predictor of mortality even in the presence of an implantable cardioverter defibrillator (ICD). Recurrent VT could be slower, below the detection threshold rate of the ICD and cause aggravation of heart failure. Those selected for a repeat ablation tended to be more sick and likely to develop VT storm.

## Justification of Class II indication for ICD in Brugada syndrome in Japanese study

Spontaneous type 1 Brugada ECG with history of syncope likely to be due to ventricular tachyarrhytmia is a Class IIa indication for the implantation of an ICD as per HRS/EHRA/APHRS Expert Consensus Statement 2013. Class IIb recommendation for ICD implantation was given for those with either a spontaneous or drug induced type 1 Brugada ECG and inducible ventricular fibrillation (VF) on programmed electrical stimulation. A Japanese multicenter study [5] has further validated these Class II indications by evaluating over two hundred patients with Brugada syndrome and these features. These were Brugada syndrome patients without a previous cardiac arrest. It was noted during follow up that cardiac events occurred more often in those with Class IIa indication than in those with Class IIb indication. This is presumably because syncope considered to be due to ventricular tachyarrhythmia has been recognized as in important risk factor for fast VT, VF and sudden cardiac death in several studies. In the present study, Class IIa indication conferred an intermediate risk of cardiac events, 2.2% per year while Class IIb indication carried a risk of only 0.5% per year.

## Figures and Tables

**Table 1:**
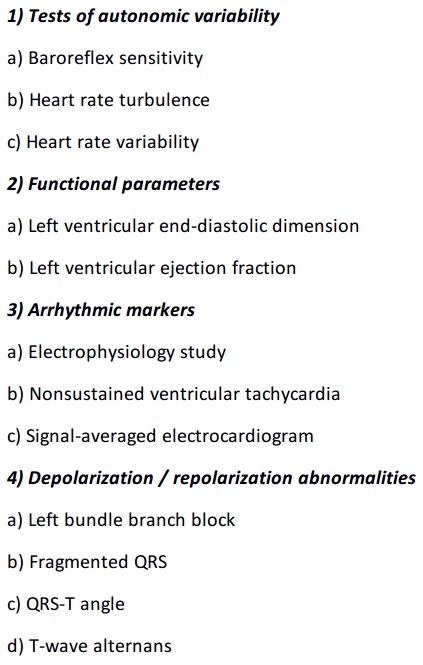
Parameters evaluated for risk stratification
